# Supplementary material for: Overexpression of a Grape WRKY Transcription Factor VhWRKY44 Improves the Resistance to Cold and Salt of Arabidopsis thaliana
Source: Int J Mol Sci. 2024 Jul 6;25(13):7437. doi: 10.3390/ijms25137437 (PMC11242199; doi:10.3390/ijms25137437)
Supplement: Supplementary file 1 [file ijms-25-07437-s001.zip › ijms-3040495-supplementary.pdf]

Supplementary Table S1. List of primers used in this study.

Gene	primer sequence (5’-3’)	Purpose
pCAMBIA1300::GFP-VhWRKY44-F	GAGCTCGGTACCCGGGGATCCATGGAGATTAAAGAGTCTGAGAGG	**Vector construction**
pCAMBIA1300::GFP-VhWRKY44-R	GCCCTTGCTCACCATGTCGACTGGTTTCTCTTTATTCGTATGGG	
VhWRKY44-F	TATGGGCAGAAGGTTGTGAAGG	**gene expression analysis**
VhWRKY44-R	TTGCGCACATTGCACTTGAG	
VvActin7(XM_002282480.4)-F	ATGGTGGTGAGCGAGAGAGG	
VvActin7(XM_002282480.4)-R	CTAGAAGTCGTCAAAGAACTGTTGC	
AtNHX1(AT5G27150)-F	AGCCTTCAGGGAACCACAAT	
AtNHX1(AT5G27150)-R	CTCCAAAGACGGGTCGCATG	
AtSOS1(AT2G01980)-F	TTCATCATCCTCACAATGGCTCTAA	
AtSOS1(AT2G01980)-R	CCCTCATCAAGCATCTCCCAGTA	
AtSOS2(AT5G35410)-F	GCAAGGGAAGAAGAAGAAGT	
AtSOS2(AT5G35410)-R	TCTCCGCTACATAACTGCC	
AtSOS3(AT5G24270)-F	GAATCCATCGCTCATCAA	
AtSOS3(AT5G24270)-R	CCATTTCTTCCTCTTCACA	
AtCOR15a(AT2G42540)-F	CAACAGAGGAATCACCAGCGA	
AtCOR15a(AT2G42540)-R	CTCTGCTGTCTTGTCGTGGTGT	
AtNCED3(AT3G14440)-F	AGACAAATACGCCGAAGA	
AtNCED3(AT3G14440)-R	CATACAGGACCCTATCACG	
AtKIN1(AT3G63480)-F	TGTCAGAGACCAACAAGAATGC	
AtKIN1(AT3G63480)-R	CCGCATCCGATACACTCTTT	
AtCBF1(AT4G25490)-F	TCGGGACTTTCCAAACCG	
AtCBF1(AT4G25490)-R	CCATCTCCTTCGCCGTCAT	
AtCBF2(AT4G25470)-F	AACTCCGGTAAGTGGGTGTG	
AtCBF2(AT4G25470)-R	CGGCGTATAAATAGCCTCCA	
AtCBF3(AT4G25480)-F	TCCGGTAAGTGGGTTTGTGAG	
AtCBF3(AT4G25480)-R	AACTCGGCATCTCAAACATCG	
AtCOR47(AT1G20440)-F	GGCTGAGGAGTACAAGAACAA	
AtCOR47(AT1G20440)-R	ACAATCCACGATCCGTAACC	
AtRAB18(AT1G43890)-F	GGGAATAAAGTTGATAAGG	
AtRAB18(AT1G43890)-R	AGAAACAAACATCCGTAC	
AtActin2(AT3G18780)-F	CCCGCTATGTATGTCGC	
AtActin2(AT3G18780)-R	AAGGTCAAGACGGAGGAT	

Note: Underline indicates respectively the cleavage sites of Sal I and BamH I.
